# On the Free Use of Opium in Colic

**Published:** 1816-05

**Authors:** 


*For the London Medical and Physical Journal.*

*On the free Use of Opium in Colic;* by an OLD CORRESPONDENT.

PERMIT me, through the medium of your valuable miscellany, to offer a few remarks on the advantages resulting from the FREE use of Opium in Colic. It having fallen to my lot to witness several cases of this description, I can speak with the greatest confidence on the good effects of this practice. Many practitioners are timid in this respect, from a fear lest the effects of the opium should increase the constipation, which always constitutes such an alarming feature of this disease; but, when it is considered that the source of this painful disorder is *a spasmodic constriction of the intestinal canal*, the propriety of persisting in the use of such remedies as are calculated to relax the spasm (of which the warm-bath and the internal exhibition of opium are the principal) will readily appear. I can venture to say, from confirmed experience on this subject, that it is perfectly fruitless to attempt to overcome the constipation by cathartics (which seem rather to increase the disposition to inflammation), until the spasmodic stricture is previously taken off by remedies suited to that intention. Wherever, therefore, the disease is correctly ascertained to depend on this cause, I would advise that opium should be given *to any extent that may be necessary.* I have known three grains given three or four times in an hour, in very urgent cases, with great advantage, until the spasm is overcome, which will be known by the cessation of pain, when it is always proper to exhibit a purge—and one of very moderate strength will, in this state, generally answer the purpose.

*March* 13, 1816.

